# Proteins in the Nutrient-Sensing and DNA Damage Checkpoint Pathways Cooperate to Restrain Mitotic Progression following DNA Damage

**DOI:** 10.1371/journal.pgen.1002176

**Published:** 2011-07-14

**Authors:** Jennifer S. Searle, Matthew D. Wood, Mandeep Kaur, David V. Tobin, Yolanda Sanchez

**Affiliations:** 1 Department of Pharmacology and Toxicology, Norris Cotton Cancer Center, Dartmouth Medical School, Hanover, New Hampshire, United States of America; 2 Department of Molecular Genetics, Biochemistry, and Microbiology, University of Cincinnati College of Medicine, Cincinnati, Ohio, United States of America; National Institute of Diabetes and Digestive and Kidney Diseases, United States of America

## Abstract

Checkpoint pathways regulate genomic integrity in part by blocking anaphase until all chromosomes have been completely replicated, repaired, and correctly aligned on the spindle. In *Saccharomyces cerevisiae*, DNA damage and mono-oriented or unattached kinetochores trigger checkpoint pathways that bifurcate to regulate both the metaphase to anaphase transition and mitotic exit. The sensor-associated kinase, Mec1, phosphorylates two downstream kinases, Chk1 and Rad53. Activation of Chk1 and Rad53 prevents anaphase and causes inhibition of the mitotic exit network. We have previously shown that the PKA pathway plays a role in blocking securin and Clb2 destruction following DNA damage. Here we show that the Mec1 DNA damage checkpoint regulates phosphorylation of the regulatory (R) subunit of PKA following DNA damage and that the phosphorylated R subunit has a role in restraining mitosis following DNA damage. In addition we found that proteins known to regulate PKA in response to nutrients and stress either by phosphorylation of the R subunit or regulating levels of cAMP are required for the role of PKA in the DNA damage checkpoint. Our data indicate that there is cross-talk between the DNA damage checkpoint and the proteins that integrate nutrient and stress signals to regulate PKA.

## Author Summary

Previous studies showed that phosphorylation of a subset of regulatory (R) subunits of the cAMP-dependent protein kinase (PKA) occurred under conditions that down-regulate global PKA activity, including growth on non-fermentable carbon sources. However, the role of the phosphorylation sites has not been elucidated. Addition of glucose to cells growing on a non-fermentable carbon source causes a transient increase of cAMP and PKA activity, which drives cells into S phase. A second peak in cAMP was proposed to restrain mitosis if the daughter cell had not reached an appropriate size. We identified a role for PKA in restraining mitosis following DNA damage. Here we provide evidence of cross-talk between the DNA damage checkpoint and PKA by phosphorylation of the R subunit. The R subunit phosphorylation sites and cAMP are necessary for the role of PKA following DNA damage. We propose that activation of PKA in response to DNA damage occurs in two steps: the phosphorylation of a subset of R subunits, probably to allow localized activation of these complexes, and cAMP to activate PKA. Our work suggests that the checkpoint and nutrient-sensing pathways share a signaling node to restrain mitosis following nutrient-induced rapid transition through the cell cycle and DNA damage.

## Introduction

Progression through mitosis in yeast requires the ordered destruction of two inhibitors: securin or Pds1 and the mitotic or B type cyclin, Clb2. The inhibitors are ubiquitinated and targeted for destruction by the anaphase promoting complex or cyclosome (APC/C) in conjunction with the specificity factors Cdc20 and Hct1/Cdh1 [1]–[3]. Destruction of Pds1 allows the separase Esp1 to cleave the cohesins to allow anaphase [4], [5]. Exit from mitosis and the establishment of pre-replication complexes requires the inactivation of the mitotic cyclin dependent kinase (Cdk) complex, achieved in part by the APC/C-mediated proteolysis of the mitotic cyclins, including Clb2, and by increased levels of the Cdk inhibitor Sic1 [6]–[9].

During anaphase the separase Esp1 triggers the release of Cdc14 from the nucleolus by modulating the phosphorylated status of its interacting partner Net1 via regulation of the phosphatase PP2A^Cdc55^ [10]–[14]. When Net1 is phosphorylated Cdc14 is released and can exit the nucleolus to dephosphorylate Cdk1 substrates such as Hct1/Cdh1, Sic1, Pds1. Cdc14 release and dephosphorylation of its targets are important for progression through anaphase and for activation of the mitotic exit network (MEN) [3], [7], [15]–[18].

Accurate transmission of chromosomes to each daughter cell requires that cells block anaphase until all chromosomes have been completely replicated and correctly aligned on the spindle. Similarly, cells that have incurred DNA damage in late S phase or G2 repair the damage before they progress through mitosis. In *S. cerevisiae*, DNA damage and mono-oriented or unattached kinetochores trigger checkpoint pathways that bifurcate to regulate both the metaphase to anaphase transition and mitotic exit [19]–[23]. In addition, several stress-activated pathways including the cAMP dependent protein kinase (PKA) pathway have been identified in *S. cerevisiae* that inhibit mitosis by targeting proteins involved in mitotic progression [24]–[28].

The sensor-associated kinase, Mec1 is activated in response to DNA damage and together with a signal amplifier, Rad9, phosphorylates two downstream kinases, Chk1 and Rad53 [29]. Activation of Chk1 prevents anaphase by preventing the destruction of securin [19], [30]; and activation of Rad53 causes inhibition of the mitotic exit network, helps to prevent securin destruction [31], [32], and also prevents segregation of damaged chromosomes by restricting spindle elongation [33].

We showed that PKA had a role in the DNA damage checkpoint. PKA supported mitotic arrest by regulating the phosphorylation of Cdc20 and helped maintain high levels of the mitotic inhibitors, securin (Pds1) and Clb2. In addition, we showed that phosphorylation of Cdc20 was both Mec1- and PKA-dependent, and that overexpression of PKA catalytic subunits partially rescued the checkpoint defect of *mec1-21* cells [34]. In the work described here, we set out to identify the mechanism by which PKA is regulated in response to DNA damage. PKA in its inactive form is a tetramer consisting of two catalytic and two regulatory subunits. In yeast three genes encode the catalytic subunits, *TPK1*, *TPK2* and *TPK3* and one gene encodes the R subunit, *BCY1* [35]–[37]. R subunit interaction with cAMP causes the tetramer to disassociate rendering the catalytic subunits active. Thus, activation of PKA is regulated by increasing the intracellular levels of cAMP. At least two other signaling pathways in yeast regulate a transient increase in cAMP levels. The Ras pathway, which can be stimulated by intracellular acidification and the glucose signaling pathway (reviewed in [38]). The glucose kinase, Hxk2, is required for the transient increase in cAMP levels in both the Ras and glucose signaling pathways [39].

Regulation of PKA can also occur via post-translational modification, protein interactions and sub-cellular localization of the catalytic or R subunits. These mechanisms of regulation have been more thoroughly studied in metazoan cells in which phosphorylation of the regulatory subunit localizes the complex to a specific sub-cellular compartment via interaction with proteins called A protein Kinase Anchoring Proteins (AKAPs). The AKAPs help mediate the specificity of PKA signaling in the cell [40]. In yeast the R subunit is phosphorylated when cells are deprived of glucose and in response to heat shock or addition of calcium to the media [41], [42]. The Yak1 kinase is required for the phosphorylation of the R subunit when the cells are grown in a non-fermentable carbon source [41], and Mck1, a GSK-3 kinase, is required for the phosphorylation of the R subunit in response to heat shock [42]. This phosphorylation is required for re-distribution of the R subunit from the nucleus to both the nucleus and cytoplasm [42]. In addition, Zds1 and Zds2 have been implicated in the regulation of the localization of the R subunit to the cytoplasm in response to the same stresses that lead to R subunit phosphorylation [41], [42].

Here we show that the checkpoint regulated phosphorylation of the R subunit of PKA has a role in restraining mitosis following DNA damage, suggesting that there is cross-talk between the DNA damage checkpoint and the PKA pathways. In addition we found that proteins that regulate the phosphorylation of the R subunit, and proteins that regulate the levels of intracellular cAMP are required for the role of PKA in the DNA damage checkpoint.

## Results

### The R subunit is phosphorylated in response to DNA damage

We have previously shown that the PKA pathway plays a role in blocking securin and Clb2 destruction following DNA damage [34]. Our findings also suggested that Mec1 and PKA are in the same signaling pathway and that PKA was likely acting downstream of Mec1 in response to DNA damage.

**Figure 1:**
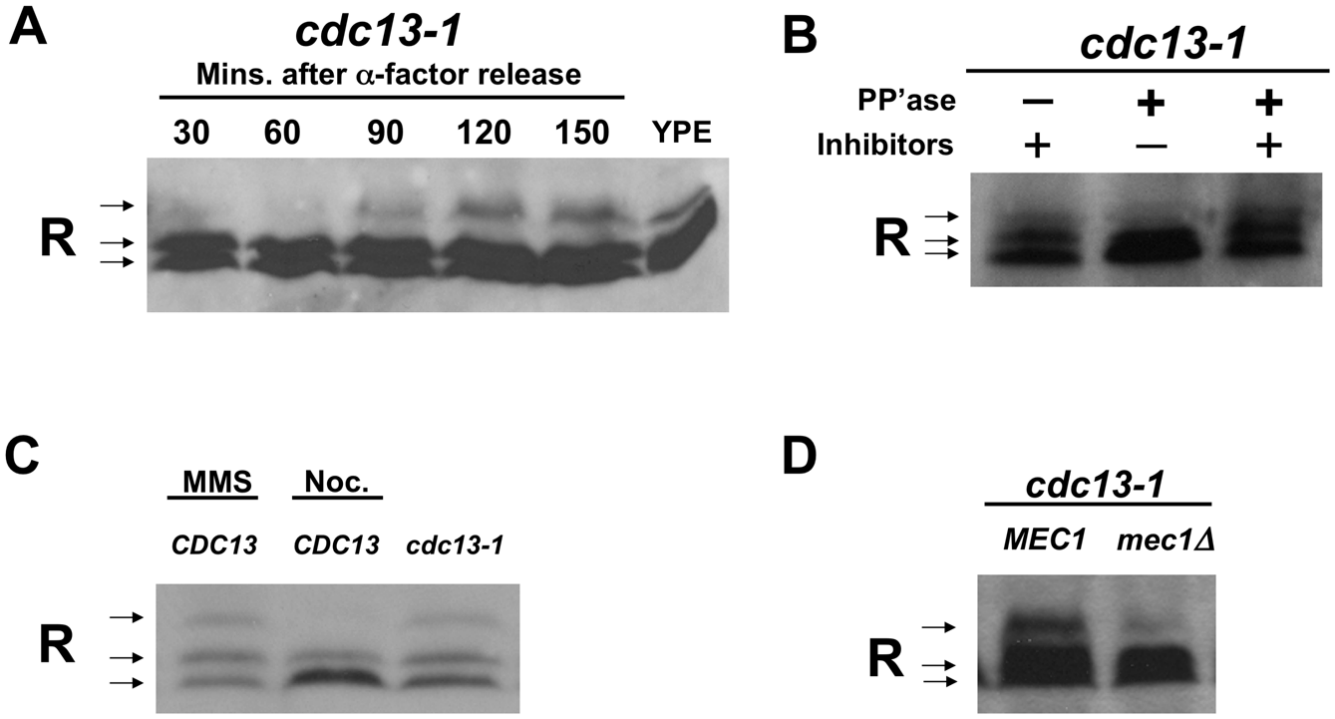
The R subunit is phosphorylated in response to DNA damage in a Mec1-dependent manner. A) *cdc13-1* cells were synchronized in α-factor and released at 32°C. Cells were collected at indicated timepoints and lysed in TCA. For the YPE sample, WT cells were grown in YP media containing ethanol overnight and the cells were lysed in TCA. Proteins were separated on a 10% polyacrylamide SDS gel. Western analysis was used to detect the R subunit using an anti-Bcy1 (R subunit) antibody. For loading control see Figure S3A. B) *cdc13-1* cells were grown at 22°C and the temperature was raised to 32°C for 120 min., cells were collected and TCA precipitated protein extracts were prepared. After re-solubilizing the TCA precipitated protein by boiling, the R subunit was isolated by immunoprecipitation and treated with alkaline phosphatase with or without phosphatase inhibitors as indicated. Separation and detection of the R subunit was carried out as described above. C) WT and *cdc13-1* cells were grown at 22°C and raised to 32°C for 2 hours. 0.1% MMS, 10 µg/ml nocodazole were added to WT as indicated for the 2 hours the cells were at 30°C. Cells were lysed and proteins detected as in (A). Loading control Figure S3B. D) *cdc13-1* and *cdc13-1 mec1Δ* cells were grown and treated as in (B), and detection of R subunit was carried out as in (A). Loading control Figure S3C, replicate experiments Figure S4.

PKA can be regulated by many mechanisms including regulation of cAMP levels, localization of the PKA holoenzyme or catalytic subunits, interactions with other proteins, and phosphorylation of the catalytic and R subunits [38], [43]. We first set out to determine whether the R subunit was phosphorylated in response to DNA damage, and whether this phosphorylation was dependent on Mec1. The *cdc13-1* allele was used to activate the DNA damage checkpoint that blocks mitotic progression [44]. Growth of *cdc13-1* cells at a restrictive temperature results in the inactivation of the telomere binding protein, Cdc13, which causes single stranded DNA at the telomeres. This single stranded DNA is recognized as DNA damage in G2/M, and the DNA damage checkpoint is activated, blocking anaphase and mitotic exit [44], [45]. In protein extracts from synchronized cells grown at the restrictive temperature for *cdc13-1*, a slower migrating form of the R subunit was detected by western analysis at a time when the DNA damage signal was present (Figure 1A). A similar slower migrating form of the R subunit was detected in protein extracts from cells growing in YP ethanol as previously shown [41].

In order to show that the slower migrating form of the R subunit was due to phosphorylation, the R subunit was isolated by immuno-precipitation from extracts prepared from cells with a DNA damage signal. The immunoprecipitated complexes were treated with phosphatase in the presence or absence of phosphatase inhibitors. Treatment with alkaline phosphatase resulted in a loss of the slower migrating form of the R subunit, while the slower migrating form of the R subunit was maintained when the immunoprecipitated-complexes were treated with both phosphatase and phosphatase inhibitors (Figure 1B). These results indicated that the R subunit was modified by phosphorylation following activation of the DNA damage checkpoint.

The R subunit was also phosphorylated in cells grown at 30°C (in the absence of heat shock) and treated with the DNA damaging agent, methylmethane sulfonate (MMS) (Figure 1C). Pre-anaphase arrest due to activation of the spindle checkpoint by treatment with nocodazole (in the absence of a DNA damage signal) did not result in R subunit phosphorylation (Figure 1C). This finding indicated that phosphorylation of the R subunit in *cdc13-1* cells was not due to a cell cycle position effect and that activation of the spindle checkpoint does not result in R subunit phosphorylation.

The R subunit was not phosphorylated in *cdc13-1* cells lacking the upstream checkpoint kinase, Mec1, (Figure 1D and Figure S4). This result suggested that Mec1 is required for the phosphorylation of the R subunit in response to DNA damage and supports the hypothesis that Mec1 regulates PKA when there is a DNA damage signal.

### Mck1 is required for R subunit phosphorylation following DNA damage and has a role in the DNA damage checkpoint

**Figure 2:**
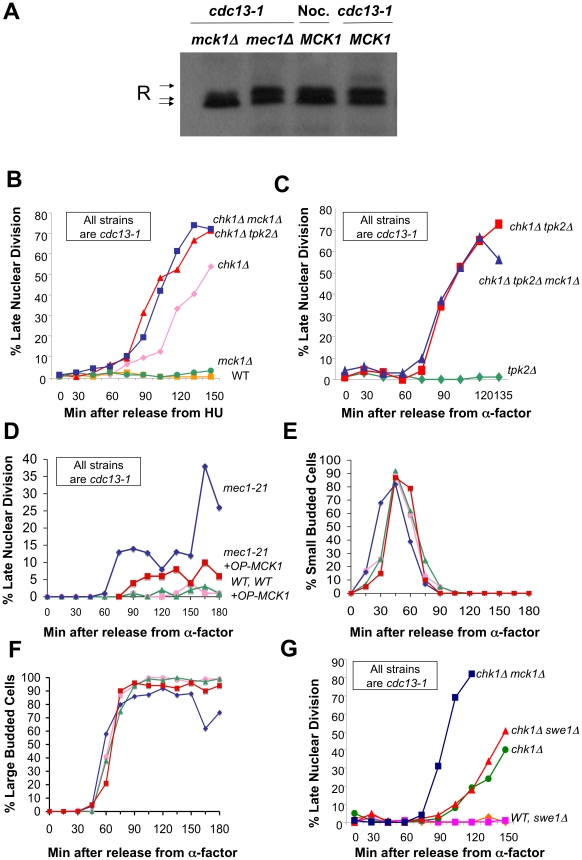
Mck1 is required for R subunit phosphorylation and participates in restraining anaphase following DNA damage. A) Phosphorylation of the R subunit in response to DNA damage is dependent on Mck1. *cdc13-1 MCK1* (WT), *cdc13-1 mck1Δ* and *cdc13-1 mec1Δ* cells were raised to 32°C for 120 min. Nocodazole was added to *MCK1* cells for 120 min. Cells were lysed, and the R subunit was detected as described in Figure 1. Loading control for (A) in Figure S5A. Replicate of experiment in (A) is shown in Figure S5B. B) *cdc13-1*, *cdc13-1 chk1Δ*, *cdc13-1 mck1Δ*, *cdc13-1 chk1Δ mck1Δ*, and *cdc13-1 chk1Δ tpk2Δ*, cells were arrested in S-phase by addition of HU (at 22°C), the temperature was raised for 60 min and the cells were released into the cell cycle at 32°C. Cells were collected at the indicated time points, fixed in ethanol, and stained with DAPI to visualize the DNA and nuclear morphology. The number of cells that exhibited a late mitotic phenotype were counted. These strains were part of experiment in Figure S1. C) *cdc13-1 tpk2Δ*, *cdc13-1 chk1Δ tpk2Δ* (same as in Figure 5C) and *cdc13-1 chk1Δ tpk2Δ mck1Δ* cells were arrested in G1 with α-factor and released into the cell cycle at 32°C. Samples were taken at indicated times and analyzed as in (B). D) *cdc13-1* and *cdc13-1 mec1-21* cells transformed with either the high-copy vector pRS425 or *MCK1* in pRS425 (OP *MCK1*) were grown at 22°C and arrested in G1 by addition of α-factor in YPD pH 3.9. The temperature was raised to 32°C and the cells were released from G1 into the cell cycle at 32°C in YPD pH 3.9. Cells were collected at the indicated time points and analyzed as in (B). Replicate experiments for (D–F) are shown in Figure S6A–S6D. E) Cells from (D) were monitored for the appearance of small buds. F) Cells from (D) were scored for the time it took to reach large budded state with an undivided nucleus. G) *cdc13-1* (WT), *cdc13-1 chk1Δ*, *cdc13-1 swe1Δ*, *cdc13-1 chk1Δ swe1*, *cdc13-1 chk1Δ mck1Δ* cells were grown, arrested, and released as in (D).

The GSK3 kinase Mck1 was found to be required for the phosphorylation of the R subunit in response to heat shock [42]. We recapitulated these findings in our lab and found that R subunit phosphorylation in response to heat shock was dependent on Mck1 but not on the DNA damage kinase Mec1 (data not shown). Thus, the requirement for Mec1 for R subunit phosphorylation was specific to a DNA damage signal. We next examined whether Mck1 regulated the phosphorylation of the R subunit in the DNA damage checkpoint. R subunit proteins from *cdc13-1* or *cdc13-1 mck1Δ* cells raised to the non-permissive temperature of 32°C for *cdc13-1* were analyzed by Western analysis. No phosphorylated R subunit was detected in *cdc13-1* cells lacking *MCK1* (Figure 2A). Our data indicate that Mck1 responds to different signals to regulate R subunit phosphorylation and that in the case of DNA damage the signal required the sensor-associated kinase Mec1.

We previously showed that PKA and Chk1 work in parallel pathways to inhibit mitotic progression in the DNA damage checkpoint response [34]. To determine whether Mck1 has a role in the DNA damage checkpoint, we examined whether deletion of *MCK1* removed the remaining DNA damage-induced mitotic delay in cells lacking *CHK1*. Because PKA pathways have a role in regulating the G1/S transition, we released the cells into the *cdc13-1* induced checkpoint from an hydroxyurea (HU) arrest (which synchronizes the cells in late S-phase) and monitored their progression through mitosis. The number of cells that had failed in the checkpoint-mediated arrest was determined by scoring the number of cells that exhibited a late mitotic phenotype (separated nuclei) in the presence of a DNA damage signal. *cdc13-1* cells lacking *MCK1* or *TPK2* were proficient in the checkpoint arrest and remained arrested throughout the experiment (Figure 2B and data not shown), however, *cdc13-1 chk1Δ* cells lacking *MCK1* or *TPK2* exhibited more cells with a late mitotic phenotype at earlier time points than the *cdc13-1 chk1Δ* cells (Figure 2B). These results indicated that Mck1 had a role in the checkpoint-mediated arrest similar to the role of Tpk2.

To determine whether Mck1 was acting in the same pathway as PKA to restrain mitosis following DNA damage, we tested whether deletion of *MCK1* would further enhance the checkpoint defect of *cdc13-1 chk1Δ tpk2Δ* cells. Cells were released into the cell cycle from an α-factor induced G1 arrest. Only cells lacking *TPK2* were used in this experiment to eliminate any differences in cell cycle entry due to role of PKA in the G1/S transition. *cdc13-1 chk1Δ* cells lacking *TPK2* failed in the checkpoint with the same kinetics as *cdc13-1 chk1Δ* cells lacking both *TPK2* and *MCK1* (Figure 2C), suggesting that Mck1 and Tpk2 were acting in the same pathway following DNA damage.

Because Mck1 and Mec1 were required for the phosphorylation of the R subunit following DNA damage, and Mck1 had been previously identified as a high-copy suppressor of the lethality associated with deletion of *RAD53* [46], we wanted to determine whether Mck1 was acting downstream of Mec1 to restrain mitosis following DNA damage. *cdc13-1* or *cdc13-1 mec1-21* cells containing an empty vector or *MCK1* on a multi-copy vector were released from G1 (α-factor block) at 32°C and the cells were stained with DAPI to determine the number of cells exhibiting late mitotic phenotype at the indicated time points. Overexpression of Mck1 partially alleviated the checkpoint defect of *mec1-21* cells following DNA damage (Figure 2D), suggesting that Mck1 was acting downstream of Mec1 to restrain mitosis following DNA damage. Overexpression of *MCK1* did not delay entry into S phase (appearance of small budded cells) (Figure 2E, and Figure S6B). Cells with a high copy plasmid encoding *MCK1* reached large budded state with an undivided nucleus with the same kinetics as vector controls (Figure 2F and Figure S6C). This was the case whether the cells were released from the G1 block into YPD or minimal media (Figure S6E–S6H).

Mck1 has been shown to play a role in restraining the G2/M cell cycle transition through activation of Swe1, a kinase that negatively regulates Cdk1 [47]. To test whether Mck1 was acting in the Swe1 pathway to restrain entry into mitosis we deleted *SWE1* in a *chk1Δ* mutant and found that *SWE1* deletion did not enhance the checkpoint defect of a *chk1Δ* cell (Figure 2G). These findings suggested that the role of Mck1 following DNA damage was to block mitotic progression, and not to block mitotic entry via Swe1.

### Phosphorylation of the R subunit is required for the role of PKA in the DNA damage checkpoint

**Figure 3:**
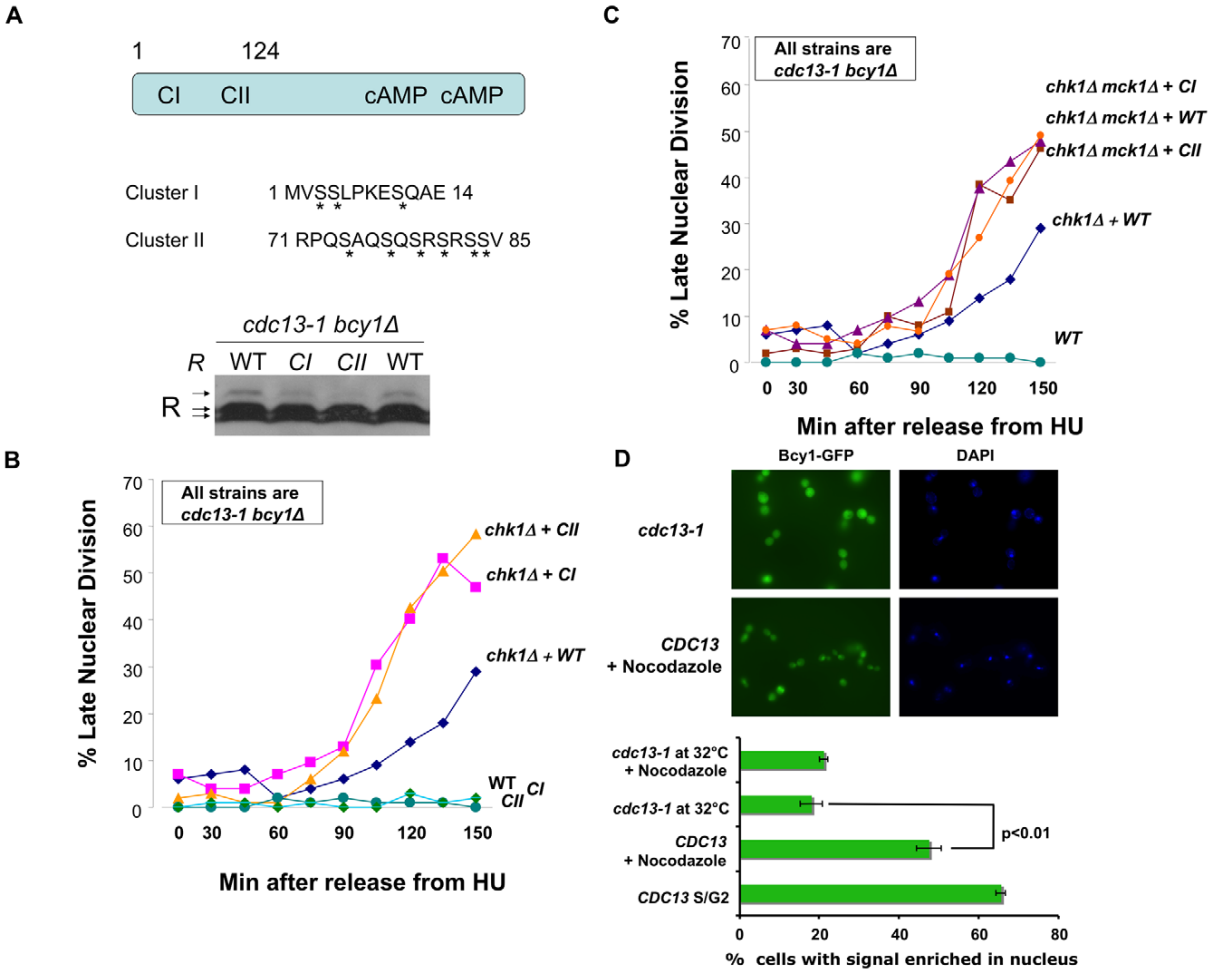
R subunit phosphorylation defective mutants enhanced the DNA damage checkpoint defect of *chk1Δ* cells. A) TOP: R subunit (Bcy1) showing position of Cluster I and II on the N-terminus and the serines changed to alanines (asterisks) in the Cluster I and Cluster II mutants. BOTTOM: *cdc13-1 bcy1Δ*+*BCY1*, *cdc13-1 bcy1Δ*+*bcy1CI*, and *cdc13-1 bcy1Δ*+*bcy1CII* cells were grown at 22°C and the temperature was raised to 32°C for 120 min. Cells were collected, lysed and the proteins were analyzed as in Figure 1A. B) All strains are in *cdc-13-1 bcy1Δ* background: *cdc13-1 bcy1Δ*+*BCY1 (WT)*, *cdc13-1 bcy1Δ*+*bcy1CI (CI)*, *cdc13-1 bcy1Δ*+*bcy1CII (CII)*, *cdc13-1 chk1Δ bcy1Δ+CY1*, *cdc13-1 chk1Δ bcy1Δ+bcy1CI*, and *cdc13-1 chk1Δ bcy1Δ+bcy1CII* cells were arrested in S-phase by addition of HU to the media. The temperature was raised to 32°C for 60 min and the cells were released into the cell cycle at 32°C. Aliquots were taken at the indicated times and each sample was analyzed as in Figure 2B. C) *cdc13-1 bcy1Δ+BCY1*, *cdc13-1 chk1Δ bcy1Δ+BCY1*, *cdc13-1 bcy1Δ chk1Δ mck1Δ+BCY1*, *cdc13-1 bcy1Δ chk1Δ mck1Δ+bcy1CI*, and *cdc13-1 bcy1Δ chk1Δ mck1Δ+bcy1CII* cells were analyzed as in Figure 2B. D) WT *BCY1-GFP* cells and *cdc13-1 BCY1-GFP* cells were grown at 22°C arrested in G1 with α-factor and released into the cell cycle at 32°C in the presence or absence of 10 µg/ml nocodazole. Localization of Bcy1-GFP in WT *BCY1-GFP* cells in late S/G2 was scored 50 min after release when the majority of cells were large budded. Bcy1-GFP localization was scored in the remainder of the cells 120 min. after release. To visualize GFP expression, cells were spotted on slides pre-coated with polylysine (Poly-L-lysine) and allowed to dry. GFP expression was analyzed using a Zeiss LSM 510 Meta confocal microscope. Graph represents average of three experiments. *WT* = Construct expressing *BCY1*, *CI* = Construct expressing *bcy1CI*, *CII* = Construct expressing *bcy1CII*. Replicate experiment shown in Figure S2.

Two serine rich clusters on the R subunit were shown to be required for the phosphorylation and cytoplasmic localization of the R subunit in response to heat shock and growth in non-fermentable carbon sources [41] (Figure 3A). However, mutation of cluster I or cluster II serines did not compromise R subunit function in response to carbon source or heat shock responses [41]. To determine whether these sites were phosphorylated in response to DNA damage, protein from cells given a DNA damage signal expressing wild type R subunit or the R subunit with serines in cluster I or cluster II [41] mutated to alanines were analyzed by western blot. The wild type R subunit was phosphorylated under these conditions but the phosphorylation was greatly reduced when the serines in cluster I or II were changed to alanine (Figure 3A). These results suggested that the R subunit is phosphorylated on serine residues located in both clusters I and II.

Since Mck1 is both required for the R subunit phosphorylation and has a role in the DNA damage checkpoint similar to PKA, we hypothesized that the role of PKA in the DNA damage checkpoint required phosphorylation of the R subunit on cluster I and cluster II serines. Therefore, phosphorylation defective mutants should have the same phenotype as deletion of a PKA catalytic subunit. *cdc13-1 bcy1Δ* or *cdc13-1 chk1Δ bcy1Δ* cells expressing wild type *BCY1* or *bcy1CI* or *bcy1CII* mutants were released into the cell cycle from an HU-mediated arrest in late S-phase into a DNA damage signal, and the number of cells exhibiting a late mitotic phenotype were counted. Expression of the phosphorylation defective R subunit mutants enhanced the checkpoint defect of a *chk1Δ* cell to the same extent as deletion of a PKA catalytic subunit (Figure 3B). Furthermore, expression of the phosphorylation defective R subunit mutants did not further enhance the checkpoint defect of cells lacking both *CHK1* and *MCK1* (Figure 3C). These results suggested that phosphorylation of the serines in cluster I and II play a role in the DNA damage checkpoint and that the role of Mck1 in the DNA damage checkpoint is via regulation of the R subunit by phosphorylation on one or more of the serine residues in clusters I and II.

### Re-localization of the R subunit following DNA damage

It was previously reported that R subunit undergoes re-localization to the cytoplasm upon heat shock and that this shift in localization was dependent on serine residues in clusters I and II [42]. Based on our observation that the phosphorylation of cluster I and II serines is important for the mitotic delay following DNA damage, we hypothesized that the DNA damage signal would alter the localization of the R subunit. To address this, the localization of GFP-tagged Bcy1 was analyzed by microscopy in WT and *cdc13-1* cells. The number of cells with nuclear accumulation of Bcy1-GFP was scored under asynchronous growth conditions, DNA damage, heat shock or by arresting cells in M phase with nocodazole. Upon induction of DNA damage, we observed a shift in the localization of the R subunit from the nucleus into the cytoplasm (Figure 3D and Figure S2). This re-localization was similar to that observed in cells following heat shock (Figure S2). As previously reported, asynchronous or nocodazole treated WT cultures had a high percentage of cells with nuclear Bcy1. These data suggest that the activation of the DNA damage checkpoint, as elicited by a *cdc13-1* mutant, promotes the export of the R subunit from the nucleus into the cytoplasm.

### Zds2 plays a role in the DNA damage checkpoint

**Figure 4:**
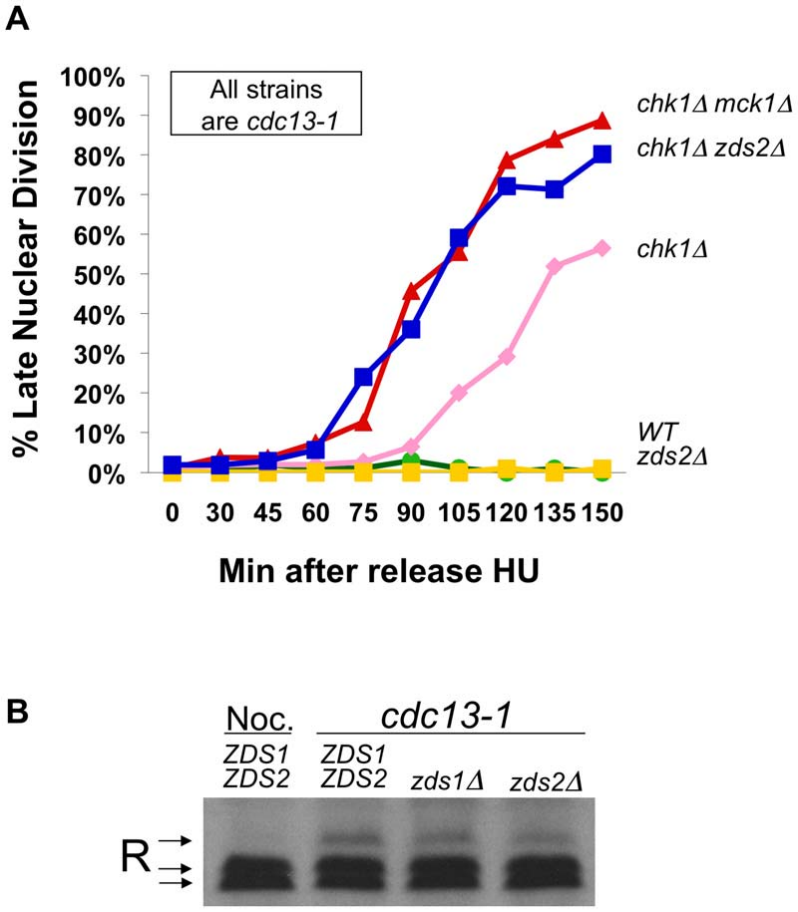
Zds2 has a role in the DNA damage checkpoint. A) *cdc13-1*, *cdc13-1 chk1Δ*, *cdc13-1 zds2Δ*, *cdc13-1 chk1Δ zds2Δ*, and *cdc13-1 chk1Δ mck1Δ* cells were arrested in S-phase using HU and released into the cell cycle at 32°C as in Figure 2B. Aliquots from each culture were taken at indicated times following release into the cell cycle, fixed and analyzed as in Figure 2B. Replicate experiment is shown in Figure S1B. B) TCA precipitated proteins from WT cells treated with nocodazole for 120 minutes, and TCA precipitated proteins from *cdc13-1*, *cdc13-1 zds1Δ*, and *cdc13-1 zds2Δ* cells grown at 32°C for 120 minutes and proteins were analyzed as in Figure 1A.

Two proteins, Zds1 and Zds2, have been implicated in regulating the cytoplasmic localization of the R subunit in the same conditions in which the R subunit is phosphorylated. Zds1 was required for the cytoplasmic localization of the R subunit in glucose restricting conditions [41], and Zds1 and its homologue, Zds2, were required for cytoplasmic localization in response to heat shock and addition of extra-cellular calcium [42]. To test whether Zds1 or Zds2 had a role in the DNA damage checkpoint, we analyzed the rate at which *cdc13-1*, or *cdc13-1 chk1Δ* cells lacking *ZDS1*, *ZDS2*, or *TPK2* failed in the checkpoint mediated arrest by scoring the number of cells exhibiting a late mitotic phenotype. *cdc13-1*, *cdc13-1 zds1Δ* and *cdc13-1 zds2Δ* cells were proficient in restraining mitosis, deletion of *ZDS1* delayed the rate at which *chk1Δ* cells failed in the checkpoint mediated arrest (Figure S1A). This result was not surprising as cells lacking Zds1 have a delay in G2 and mitosis under normal growth conditions [47]. However *cdc13-1 chk1Δ* lacking *ZDS2*, *MCK1* or *TPK2* exhibited more cells with a late mitotic phenotype at earlier time points than *cdc13-1 chk1Δ* (Figure 4A and Figure S1B). The rate at which *cdc13-1 chk1Δ* lacking *ZDS2*, *MCK1* or *TPK2* failed in the checkpoint was identical, suggesting that Zds2 has a role in the checkpoint similar to the role of Tpk2 and Mck1. Neither Zds1 nor Zds2 was required for the phosphorylation of the R subunit following DNA damage (Figure 4B), indicating that Zds2 has a role in the checkpoint independent of the phosphorylation of the R subunit.

### cAMP signaling and Hxk2 are both required for the PKA role in the DNA damage checkpoint-induced delay in mitosis

**Figure 5:**
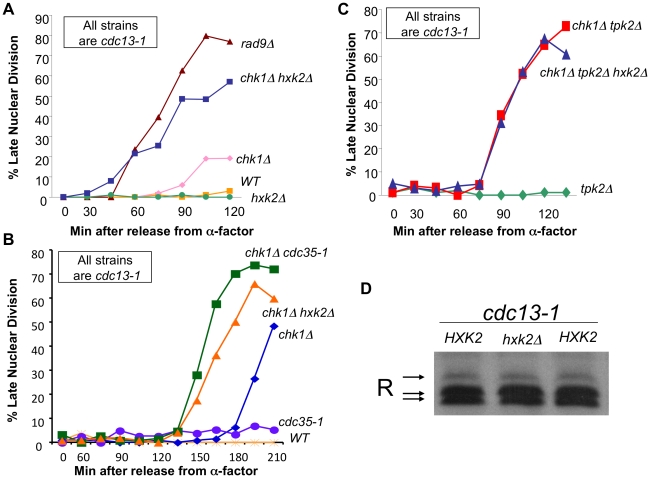
cAMP and Hxk2 are required for the role of PKA in the DNA damage checkpoint. A) *cdc13-1*, *cdc13-1 chk1Δ*, *cdc13-1 rad9Δ*, *cdc13-1 hxk2Δ*, and *cdc13-1 chk1Δ hxk2Δ* cells were arrested in G1 using α-factor and released into the cell cycle at 32°C. Aliquots were removed at indicated time points and analyzed as in Figure 2B. B) *cdc13-1*, *cdc13-1 chk1Δ*, *cdc13-1 cdc35-1*, *cdc13-1 cdc35-1 chk1Δ*, *cdc13-1 chk1Δ hxk2Δ* cells were arrested in G1 with α-factor and released into the cell cycle at 22°C. 30 minutes following release from G1 the temperature was gradually raised so that 45 minutes after release from G1 the temperature was 35°C. Aliquots of the cells were taken at the indicated times and were analyzed as in Figure 2B. C) *cdc13-1 tpk2Δ*, *cdc13-1 chk1Δ tpk2Δ*, and *cdc13-1 chk1Δ tpk2Δ hxk2Δ* cells were released from a G1 block into the cell cycle at 32°C. Aliquots of the cells were taken at indicated timepoints and analyzed as in Figure 2B. The *chk1Δ tpk2Δ hxk2Δ* cells were run along with strains in Figure 2C, therefore the control strains are the same as those shown in Figure 2C. D) *cdc13-1*, *cdc13-1 hxk2Δ* cells were grown at 22°C and the temperature was increased to 32°C for 120 minutes. The cells were lysed and the proteins analyzed as in Figure 1A.

Our genetic experiments support the existence of a signaling complex that includes phosphorylated R subunit, PKA catalytic subunit(s), and possibly Zds2. To examine whether cAMP is required to activate this complex following DNA damage, proteins involved in the regulation of cAMP signaling were deleted or mutated in *cdc13-1 chk1Δ* cells. In this way we could gain insight as to whether or not cAMP was required for the role of PKA in the DNA damage checkpoint. A *cdc35-1* mutation, which causes inactivation of adenylate cyclase when the cells are grown at the restrictive temperature [48], [49], was introduced into *cdc13-1* and *cdc13-1 chk1Δ* cells so that we could determine whether adenylate cyclase had a role in the DNA damage checkpoint. The cells were released from a G1 arrest into the cell cycle. A DNA damage signal was generated and/or adenylate cyclase was inactivated as the cells progressed through S phase by raising the temperature to 35°C. Cells were scored for nuclear division as in previous assays. Due to the timing of Cdc35 inactivation in *cdc35-1* cells, only 40% of *cdc13-1 cdc35-1* entered S phase as evidenced by budding, consequently only the large-budded fraction was scored for this strain in the later timepoints. All other strains were uniformly large budded at later timepoints. Inactivation of adenylate cyclase enhanced the checkpoint defect of the *chk1Δ* cell (Figure 5B), suggesting that cAMP is required to activate the PKA complexes that play a role in the DNA damage checkpoint.

Hexokinase 2 (Hxk2) is required for transient activation of cAMP signaling upon addition of glucose to cells growing in a non-fermentable carbon source and in cells with intracellular acidification [39]. We show here that deletion of *HXK2* exacerbated the checkpoint defect of *cdc13-1 chk1Δ* cells (Figure 5A). The *cdc13-1 chk1Δ hxk2Δ* cells failed in the checkpoint with similar kinetics as a *cdc13-1 rad9Δ* cell (Figure 5A), indicating that the majority of the checkpoint response was gone in *cdc13-1 chk1Δ hxk2Δ* cells. Deletion of *HXK2* also enhanced the checkpoint defect of *chk1Δ* cells with similar kinetics as inactivation of adenylate cyclase (Figure 5B) and did not enhance the checkpoint defect of *cdc13-1 chk1Δ tpk2Δ* cells (Figure 5C), suggesting that Hxk2 is acting in the same pathway as PKA to help restrain mitosis following DNA damage. In addition, Hxk2 was not required for phosphorylation of the R subunit following DNA damage (Figure 5D).

### Mck1, Zds1, and Zds2 play roles in the re-localization of the R subunit following DNA damage

**Figure 6:**
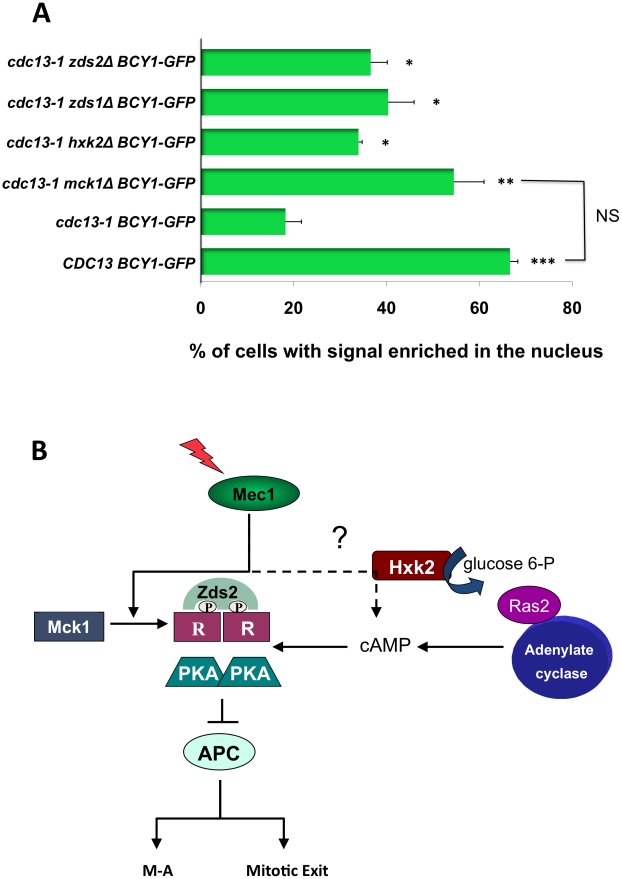
Mck1, Zds1, Zds2, and Hxk2 are required for R subunit re-localization after DNA damage. A) WT *BCY1-GFP* cells, *cdc13-1 BCY1-GFP*, *cdc13-1 mck1Δ BCY1-GFP*, *cdc13-1 hxk2Δ BCY1-GFP*, *cdc13-1 zds1Δ BCY1-GFP* and *cdc13-1zds2Δ BCY1-GFP* cells were grown at 22°C arrested in G1 with α-factor and released into the cell cycle at 32°C. Cells were observed under the microscope until large budded. Localization of Bcy1-GFP in WT *BCY1-GFP* cells in late S/G2 was scored 50 min after release when the majority of cells were large budded. Bcy1-GFP localization was scored in the remainder of the cells 120 min. after release. To visualize GFP expression, cells were spotted on slides pre-coated with polylysine (Poly-L-lysine) and allowed to dry. GFP expression was analyzed using a Zeiss LSM 510 Meta confocal microscope. Graph represents average of three experiments. Asterisks represent significance compared to *cdc13-1 GFP-BCY1*. * = p<0.05, ** = p<0.01, *** = p<0.001, NS = Not significant. All T-tests are two-tailed. B) Model, see text for details.

Both Zds1 and Zds2 have been shown to be involved in regulating the cytoplasmic localization of the phosphorylated R subunit under conditions in which the phosphorylation of the R subunit was Mck1 dependent [42]. Thus we examined whether Zds1, Zds2 and Hxk2, which did not play a role in R subunit phosphorylation following damage, could regulate PKA by causing the cytoplasmic localization of the R subunit. We also examined whether the cytoplasmic localization following DNA damage was dependent on Mck1, as it was required for the R subunit phosphorylation in this response. Localization of GFP-tagged Bcy1 was analyzed by microscopy in WT *BCY1-GFP* cells, *cdc13-1 BCY1-GFP*, *cdc13-1 mck1Δ BCY1-GFP*, *cdc13-1 hxk2Δ BCY1-GFP*, *cdc13-1 zds1Δ BCY1-GFP* and *cdc13-1 zds2Δ BCY1-GFP* cells released from a G1 block into the cell cycle at the restrictive temperature for *cdc13-1*. As shown before, G2/M *cdc13-1* cells had a significant decrease in nuclear enrichment of Bcy1-GFP signal in the nucleus, indicating cytoplasmic re-localization of the R subunit following a DNA damage signal (Figure 6A, compare nuclear enrichment of Bcy1-GFP *in CDC13* vs *cdc13-1* cells). We found that the re-localization of R subunit in G2/M cells with a DNA damage signal was completely dependent on Mck1. In fact there was no difference in the cells with damage signal lacking Mck1 (*cdc13-1 mck1Δ BCY1-GFP* ) and the cells with no damage signal *(CDC13 BCY1-GFP*). The re-localization of the R subunit was also dependent on Zds1, Zds2 and Hxk2.

These results support a model (Figure 6B) in which phosphorylation and relocalization of the R subunit as well as cAMP are both required to establish the PKA signal following DNA damage in order to help restrain mitotic progression.

## Discussion

How a holoenzyme such as PKA can interpret different stimuli to elicit a distinct cellular response is a timely question. PKA specificity of signaling in mammalian cells is regulated at least in part by phosphorylation of the catalytic or regulatory subunits, as well as interactions with specific proteins, and sub-cellular localization. Previous studies in yeast have shown that the R subunit of PKA is phosphorylated in response to various signals; however, the phosphorylation of the R subunit did not have a role in regulating responses previously associated with PKA activation [42]. In budding yeast the PKA pathway antagonizes expression of genes that are required for cells to tolerate stress from starvation, heat shock and oxidative damage [50]. Those signals and DNA damage, which lead to phosphorylation and redistribution of the R subunit, correlate with global down-regulation of PKA [50]. Therefore, phosphorylation of the R subunit could be a mechanism to allow localized activation of the holoenzyme following stress.

We have shown that the catalytic subunits of PKA have a role in restraining mitosis after DNA damage. Our data presented here indicated that phosphorylation of the R subunit in response to DNA damage could contribute to specifically target PKA to substrates involved in restraining mitosis. Previous studies showed that growing cells in non-fermentable carbon sources led to phosphorylation of the R subunit, and that addition of glucose to cells growing in a non-fermentable carbon source caused a transient increase of intracellular cAMP. The PKA activity following glucose addition drives cells into S phase and it had been proposed that PKA activity also helped restrain mitosis if the daughter cell had not reached an appropriate size [27]. Therefore we propose that the checkpoint and nutrient-sensing pathways share a signaling node to restrain mitosis under two different conditions: 1) following nutrient-induced rapid transition through the cell cycle and 2) DNA damage.

### Model for activation of PKA by the DNA damage checkpoint

Previous studies showed that growing cells in non-fermentable carbon sources led to phosphorylation of the R subunit [41], and that addition of glucose to cells growing in a non-fermentable carbon source caused a transient increase of intracellular cAMP [51]. From these data we postulated that activation of PKA in response to DNA damage is carried out in two steps: the phosphorylation of a small population of the R subunit, probably to provide the specificity of the signal, and a requirement for cAMP to activate PKA catalytic activity. We showed that in addition to the serine residues in clusters I and II of the R subunit, adenylate cyclase was also required for the DNA damage checkpoint mediated arrest that could be measured in cells lacking *CHK1*. We have identified four proteins that have a role in regulating PKA activity in the DNA damage checkpoint. These proteins were previously implicated in regulation of PKA by 1) regulating the phosphorylation of the R subunit (Mck1), 2) regulating the cytoplasmic localization of the R subunit (Zds2) [42], and 3) regulation of cAMP levels (Hxk2, Cdc35) [39]. We found that all four proteins had a similar role to that of the PKA catalytic subunits in restraining mitosis suggesting that they function in the same pathway. However only Mck1, but not Zds2 or Hxk2, was required for the phosphorylation of the R subunit.

Mck1 was originally identified and characterized by Shero *et al.*, as a high copy suppressor of a defect in chromosome segregation caused by a single base pair insertion in CDEIII, one of the three conserved DNA binding domains in the centromere of a marker chromosome fragment [52]. Disruption of Mck1 alone did not cause chromosome segregation defects but the *mck1Δ* cells were benomyl sensitive. Based on this role of Mck1, it will be interesting to determine in the future whether Bcy1 phosphorylation after DNA damage plays a role in preventing chromosome segregation defects. Based on the functions previously attributed to these proteins and the evidence presented here we propose that in response to DNA damage the R subunit is phosphorylated in a checkpoint- and Mck1-dependent fashion. The phosphorylated R subunit, which could still be associated with the catalytic subunits, is further regulated by Zds2, possibly by protein-protein interaction or sub-cellular localization. In the final step of regulation, Hxk2-regulated cAMP levels are required for activation of PKA signaling to help restrain mitosis.

Our findings also implicate a possible role for Ras2 in PKA signaling in the DNA damage checkpoint as the *cdc35-1* allele encodes a protein with a single amino acid substitution in the Ras-binding domain [49]. In fact, we recently reported that deletion of the *IRA1* and *IRA2* genes encoding negative regulators of Ras prevents cellular recovery from a *cdc13-1-*induced DNA damage induced arrest. The *ira1Δ ira2Δ* recovery defect required the PKA catalytic subunit Tpk2 and the PKA phosphorylation sites on the anaphase promoting complex specificity factor Cdc20, indicating a link between the recovery defect and PKA regulation of mitosis [53]. Our data support a model in which the R subunit phosphorylation and cAMP signaling are working together to achieve the PKA-mediated restraint of mitosis following DNA damage (Figure 6B).

### Model for Zds2 regulation of PKA following DNA damage

Zds2 may also help provide specificity to PKA signaling. We found that deletion of Zds2, but not Zds1, enhanced the checkpoint defect of *chk1Δ* cells. In fact, deletion of *ZDS1* delayed the rate at which *chk1Δ* cells failed in the checkpoint-mediated arrest (Figure S1A). Cells lacking *ZDS1* have a delay in G2 and mitosis under normal growth conditions [47]. Thus, Zds1 may play a role in restraining anaphase that cannot be uncovered due to the fact that lack of Zds1 triggers a G2 delay. Furthermore, it was previously shown that both Zds1 and Zds2 were involved in regulating the cytoplasmic localization of the phosphorylated R subunit under conditions in which the phosphorylation of the R subunit was Mck1 dependent [42]. We show here that the cytoplasmic localization of the R subunit following DNA damage requires both Zds1 and Zds2. Based on these data we cannot rule out a role for Zds1 in restraining mitosis following DNA damage.

The Cdc14 phosphatase plays a key role in reversing Cdk phosphorylation and reducing mitotic cyclin/Cdk activity required for cells to exit mitosis [7]. It was recently shown that Zds1 and Zds2 are required for the timely activation of Cdc14 during mitotic exit. Cdc14 is sequestered in the nucleolus by the inhibitory protein Net1/Cfi1 prior to anaphase. This is achieved by the role of the phosphatase PP2A^Cdc55^ in maintaining Net1/Cfi1 in the de-phosphorylated state. Separase, along with other proteins of the Cdc Fourteen Early Anaphase Release (FEAR) pathway, regulate Cdc14 release during anaphase by downregulation of PP2A^Cdc55^, which results in Net1 phosphorylation. Zds1 and Zds2 interact with PP2A^Cdc55^ and are required for the separase-mediated downregulation of PP2A^Cdc55^ to allow Cdc14 release [54]. One possible role for Zds2 (and Zds1) in restraining mitosis would be that these proteins may act as scaffolds that maintain PP2A in an active state by regulating PKA localization. In this manner PKA could be antagonizing separase in the regulation of PP2A to release Cdc14.

Little is known about how Zds1 and Zds2 regulate R subunit localization, however it is possible that Zds2 is required for the export of the phosphorylated R subunit from the nucleus, but not the PKA catalytic subunit thereby maintaining active catalytic subunits in the nucleus to help restrain mitosis. Alternatively Zds2 could act to retain the holoenzyme in the cytoplasm. Zds1 has been shown to localize to the bud neck and cortex when overexpressed [55], and complexes at the daughter spindle pole body, bud neck and daughter bud cortex have been shown to play roles in the spatio-temporal regulation of mitotic progression including regulators of MEN [56]–[58] and components of PP2A^Cdc55^ [59]. Therefore, Zds2 (and Zds1) could localize the PKA holoenzyme at multiple sites where it could play a role in regulation of mitotic exit: spindle pole body, daughter cortex or at the bud neck.

### Models for the role of cAMP during the DNA damage-induced checkpoint delay

In yeast increased cAMP due to intracellular acidification, or addition of glucose to cells growing in a non-fermentable carbon source require Hxk2 [39]. Our data show that either deletion of *HXK2* or inactivation of adenylate cyclase enhanced the checkpoint defect of *chk1Δ* cell suggesting that Hxk2 regulates cAMP levels following DNA damage. Since we do not know whether or not cAMP levels increase in response to DNA damage, there are at least two models by which Hxk2 could be regulating cAMP in the checkpoint response. In the first model we propose that a basal level of cAMP, which is maintained by Hxk2 is sufficient for the activation of PKA in the checkpoint response. Alternatively the checkpoint could lead to increased cAMP levels in the cell by regulating Hxk2 and possibly Ras2 (Figure 6B).

Our data supports a model in which the DNA damage checkpoint can regulate the PKA pathway to induce specific PKA signaling in order to phosphorylate substrates that act to restrain mitosis. We identified three proteins (Mck1, Zds2, Hxk1) that along with adenylate cyclase (Cdc35) and the R subunit (Bcy1) have a novel role in the DNA damage checkpoint via regulation of PKA. Mutation or mis-regulation of PKA subunits has been associated with chromosomal instability in cancer cells [60]. It will be interesting to determine whether PKA also participates in the response to DNA damage in mammals.

## Materials and Methods

### Strains and plasmids

Strains used in this study are listed in Table 1. Yeast strains were generated using standard genetic techniques. For deletion of *BCY*1, a DNA fragment was generated by the PCR, which contained the *URA3* gene flanked by 50 bp of sequences homologous to the 5′ and 3′-UTRs of *BCY1*. The DNA fragment was then transformed into Y300, resulting in the replacement of *BCY1* with *URA3* by homologous recombination. Gene disruption was confirmed by PCR. The *BCY1:URA3* was introduced into the strains used in these studies by crossing.

**Table 1:** Yeast strains used in these studies.

Name	Genotype	Source
Y300	MATa *ade2-1 trp1-1 ura3-1 leu2-3,112 his 3-11,15 can1-100*	[63]
Y816	As Y300 *cdc13-1*	[34]
Y818	As Y300 *cdc13-1 chk1-Δ::HIS3*	[19]
YJS50	As Y816 *rad9Δ::HIS3*	This study
YJS51	As Y816 *mec1-Δ::HIS3 GAP-RNR1:TRP1*	This study
YJS52	As Y816 *mck1* ***Δ*** *::KanMX*	This study
YJS53	As Y816 *zds1* ***Δ*** *::KanMX*	This study
YJS54	As Y816 *zds2* ***Δ*** *::KanMX*	This study
YJS55	As Y816 *hxk2* ***Δ*** *::KanMX*	This study
YJS56	As Y818 *mck1* ***Δ*** *::KanMX*	This study
YJS57	As Y818 *zds1Δ::KanMX*	This study
YJS58	As Y818 *zds2Δ::KanMX*	This study
YJS76	As Y300 *rad9Δ::HIS3*	This study
YJS10	As Y818 *tpk2Δ::KanMX*	[34]
YJS59	As YJS10 *mck1Δ::KanMX*	This study
YJS8	As Y816 *tpk2Δ::KanMX*	[34]
YJS60	As Y816 *mec1-21*+pRS425	This study
YJS61	As Y816 *mec1-21*+pJS10	This study
YJS62	As Y816 + *pRS425*	This study
YJS63	As Y816 + pJS10	This study
YJS64	As Y816 *bcy1Δ::URA3*	This study
YJS65	As YJS64 + pJS11	This study
YJS66	As YJS64 + pYCJ1	This study
YJS67	As YJS64 + pYCJ2	This study
YJS68	As Y818 *bcy1Δ::URA3*	This study
YJS69	As YJS68 + pJS11	This study
YJS70	As YJS68 + pYCJ1	This study
YJS71	As YJS68 + pYCJ2	This study
YJS72	As Y818 *hxk2Δ::KanMX*	This study
YJS73	As YJS10 *hxk2Δ::KanMX*	This study
YJS74	As Y816 *cdc35-1*	This study
YJS75	As Y818 *cdc35-1*	This study
YJS76	As Y816 *swe1Δ::URA3*	This study
YJS77	As Y818 *swe1Δ::URA3*	This study
YJS78	As Y300 *mck1Δ::KanMX*	This study
Y581	As Y300 *mec1Δ::HIS3 trp1-1::GAP-RNR1-TRP1*	[64]
Y831	As Y816 *rad53-21*	[19]
YMK54	As Y300 BCY1-GFP: HIS3MX6	This study
YMK55	As Y816 BCY1-GFP: HIS3MX6	This study
YMK58	As YJS52 BCY1-GFP: HIS3MX6	This study
YMK57	As YJS53 BCY1-GFP: HIS3MX6	This study
YMK60	As YJS54 BCY1-GFP: HIS3MX6	This study
YMK59	As YJS55 BCY1-GFP: HIS3MX6	This study

See Materials and Methods for details.

Deletion of *ZDS1*, *MCK1*, and *HXK2* was carried out by using a construct generated by the PCR which resulted in amplification of the genomic DNA surrounding the gene that had been replaced with *KanMX*. Genomic DNA from strains from the deletion strain collection (Open Biosystems) were used as template for these reactions. The *BCY1-GFP* strains were generated by crossing clone YIL033C from the GFP-fusion library (Invitrogen) into Y300 background five times followed by crossing into *cdc13-1* strain in Y300 background.

*cdc35-1* strains were generated by crossing the *cdc35-1* allele from CMY282 [49] into the Y300 strain background before crossing into *cdc13-1* strains.

Plasmids used in this study are listed in Table 2. *MCK1* including upstream and downstream sequences was amplified using the primers: CTGGATCCTCTTCCCTCTTTCCCAATT, and GCTCTAGATAAACAGCGGATCA AAGG which contained a BamHI and XbaI site respectively. The amplified sequence was ligated into a pRS425 vector that had been cut with BamHI and XbaI.

**Table 2:** Constructs used in these studies.

Name	Description	Source
pJS10	*MCK1* in pRS425	This Study
pJS11	*BCY1* in pRS415	This Study
pYCJ1	*bcy1CI* in pRS415	This Study
pYCJ2	*bcy1CII* in pRS415	This Study

See Materials and Methods for details.

pJS11 was generated by subcloning *BCY1* from pXP1 [61] into pRS425 using BamHI and HindIII. pYCJ1 was generated as described in [41] pYCJ2 was generated using a three step PCR method using pJS11 and the primers:

Reverse: CTGGCTCGAGCTTGAGCTTGAGCTGCTTGAGGTCTGGAAAATGAC

Forward CAAGCTCAAGCTCGAGCCAGAGCGGCTGTTATGTTCAAATCCCCC which generated the serine to alanine mutations described in [41]. The resulting PCR product was used to amplify the region upstream of the mutated sites using pJS11 as the template and the forward primer: CGTCCGACTTTCTTCAGTTC. The PCR product generated in the second PCR was used as the forward primer and the reverse primer: CGTCATACATGAGTCTCTTC were used to amplify the region between BspEI and BsrGI sites containing the nucleotide changes to generate the serine to alanine mutations.

The resulting PCR product replaced the fragment generated by digestion of pJS11 with BspEI and BsrGI.

### Growth conditions

Cells were grown in YPD rich medium or SC-Leu medium. When α-factor was added to synchronize cells SC-Leu or YPD at pH 3.9 were used.

### Visualization of nuclei

Cells were grown to OD_600_ = 0.5–0.8 at 22°C. Cells were synchronized in G1 using α-factor (10 µg/ml) or in late S-phase using 200 mM Hydroxyurea (HU) as indicated. Unless indicated otherwise, the temperature was raised to 32°C for 60 min. prior to release. Cells were washed and re-suspended in YPD pH 3.9 at 32°C to release the cells into the cell cycle. To stop the cells from undergoing multiple cell cycles, α-factor was added back to the cells when cells released from a G1 block entered S-phase (re-budded) or immediately upon release for cells released from a late S-phase arrest. The cells were fixed and permeabilized using 70% ethanol. Cells were re-hydrated in PBS and stained with 0.1 mg/ml 4′, 6-diamidino-2-phenylindole dihydrochloride (DAPI, Sigma, St. Louis, MO). Cells were mounted on glass slides coated with 0.1% poly-L-lysine for microscopy.

### Western analysis

Protein extracts were prepared by trichloro-acetic acid (TCA) precipitation as previously described [62]. Proteins were separated on 10% acrylamide/0.067% bis-acylamide gels, and transferred to nitrocellulose membranes. Bcy1 was detected by Western analysis using anti-Bcy1 antibody (Santa Cruz Biotechnology, Santa Cruz, CA). The immune complexes were detected by chemiluminescence (NEN).

### Phosphatase assay

Protein from *cdc13-1* cells grown to OD_600_ = 0.5 at 22°C and incubated at 32°C for 120 min. was isolated by TCA precipitation. TCA precipitated protein was re-solubilized by boiling in buffer containing 1% SDS and 10 mM Tris pH 8. Yeast lysis buffer with no SDS was used to dilute the solubilized protein solution so that the final SDS concentration was 0.1%. Bcy1 was immuno-precipitaed using anti-Bcy1 antibody (Santa Cruz) and protein-A sepharose beads (Amersham). Bcy1 bound to the beads was treated with alkaline phophatase (Boehringer Mannheim) and/or phosphatase inhibitors (1 mM sodium orthovanadate, and 1 mM sodium fluoride). Bcy1 was released from the beads by boiling in Laemmli sample buffer (4% SDS, 20% glycerol, 10% 2-mercaptoethanol, contaning bromophenol blue and 0.125 M Tris-HCl pH 6.8). Immuno-precipitated proteins were resolved and analyzed as described above.

## Supporting Information

Figure S1 Zds2 has a role in the DNA damage checkpoint. A) *cdc13-1*, *cdc13-1 chk1Δ*, *cdc13-1 zds1Δ*, *cdc13-1 chk1Δ zds1Δ* and *cdc13-1chk1Δ tpk2Δ* cells were arrested in S-phase using HU and released into the cell cycle at 32°C as in Figure 2B. Aliquots from each culture were taken at indicated times following release into the cell cycle, fixed and analyzed as in Figure 2B. B) *cdc13-1*, *cdc13-1 chk1Δ, cdc13-1 zds2Δ*, *cdc13-1 chk1Δ zds2Δ*, and *cdc13-1 chk1Δ tpk2Δ* cells were arrested in S-phase using HU and released into the cell cycle at 32°C as in Figure 2B. Aliquots from each culture were taken at indicated times following release into the cell cycle, fixed and analyzed as in Figure 2B. These two graphs represent data from one experiment, therefore the controls *cdc13-1 chk1Δ and cdc13-1 chk1Δ tpk2Δ* are the same in both graphs.
(TIF)

Figure S2 R subunit re-localization following DNA damage. WT *BCY1-GFP* cells and *cdc13-1 BCY1-GFP* cells were grown at 22°C and raised to 32°C for 2 hours. 10 µg/ml nocodazole was added to the cells as indicated for the 2 hours the cells were at 32°C. To induce heat shock, WT *BCY1-GFP* cells were grown at 22°C and raised to 37°C for 3 hours. To visualize GFP expression, cells were spotted on slides pre-coated with polylysine (Poly-L-lysine) and allowed to dry. GFP expression was analyzed using a Zeiss LSM 510 Meta confocal microscope. Graph represents average of two experiments.
(TIF)

Figure S3 Loading controls for Figure 1. A) Entire lanes for blot shown in Figure 1A to show cross-reacting bands (*) as loading controls. B) Entire lanes for blot shown in Figure 1C to show cross-reacting bands (*) as loading controls. C) Entire lanes of Western blot of Bcy1 shown in Figure 1D stained with Ponceau S to show protein loading.
(TIF)

Figure S4 Replicate experiments showing dependence of DNA damage-induced mobility shift of Bcy1 (R subunit) on the checkpoint kinase Mec1. A) Replicate experiment to that shown in Figure 1D. *cdc13-1* and *cdc13-1 mec1Δ* cells were grown and treated as in Figure 1B, and detection of R subunit was carried out as in Figure 1A. B) Replicate experiment to those shown in Figure 1D and (A). *cdc13-1* and *cdc13-1 mec1Δ* cells were grown and treated as in Figure 1B, and *CDC13* cells were incubated in Nocodazole as in Figure 1C. Detection of R subunit was carried out as in Figure 1A. C) Entire lanes for blot shown in (B) (here) to show cross-reacting bands (*) as loading controls. R = Bcy1.
(TIF)

Figure S5 Loading control for Figure 2A and replicate experiment showing dependence of DNA damage-induced mobility shift of Bcy1 (R subunit) on the kinase Mck1. A) Entire lanes for blot shown in Figure 2A to show cross-reacting bands (*) as loading controls. B) Replicate experiment to that shown on Figure 2A. *cdc13-1 MCK1* (WT), and *cdc13-1 mck1Δ* cells were raised to 32°C for 120 min. Nocodazole was added to *MCK1* cells for 120 min. Cells were lysed, and the R subunit was detected as described in Figure 1.
(TIF)

Figure S6 Mck1 overexpression partially alleviated the checkpoint defect of *mec1-21* cells following DNA damage. A) *cdc13-1* and *cdc13-1 mec1-21* cells transformed with either the high-copy vector pRS425 or *MCK1* in pRS425 (OP *MCK1*) were grown at 22°C and arrested in G1 by addition of α-factor. The temperature was raised to 32°C and the cells were released from G1 into the cell cycle at 32°C. Cells were collected at the indicated time points and analyzed as in Figure 2B. B) Cells from A were monitored for the appearance of small buds. C) Cells from A were scored for the time it took to reach large budded state with an undivided nucleus. D) *cdc13-1* and *cdc13-1 mec1-21* cells transformed with either the high-copy vector pRS425 or *MCK1* in pRS425 (OP *MCK1*) were grown at 22°C and arrested in G1 by addition of α-factor. The temperature was raised to 32°C and the cells were released from G1 into the cell cycle in YPD at 32°C. Cells were collected at the indicated time points and analyzed as in Figure 2B (Replicate of Figure 2D and part A here). E) *cdc13-1* and *cdc13-1 mec1-21* cells transformed with either the high-copy vector pRS425 or *MCK1* in pRS425 (OP *MCK1*) were grown at 22°C and arrested in G1 by addition of α-factor in SC -Leu. The temperature was raised to 32°C and the cells were released from G1 into the cell cycle at 32°C in SC -Leu. Cells were collected at the indicated time points and scored for the appearance of small budded cells. F) Cells from (E) were scored for the time it took to reach large budded state with an undivided nucleus. G) and H) Replicate of experiment shown in (E,F).
(TIF)
