# Supplementary figures and images for: Proteins in the Nutrient-Sensing and DNA Damage Checkpoint Pathways Cooperate to Restrain Mitotic Progression following DNA Damage
Source: PLoS Genet. 2011 Jul 14;7(7):e1002176. doi: 10.1371/journal.pgen.1002176 (PMC3136438; doi:10.1371/journal.pgen.1002176)

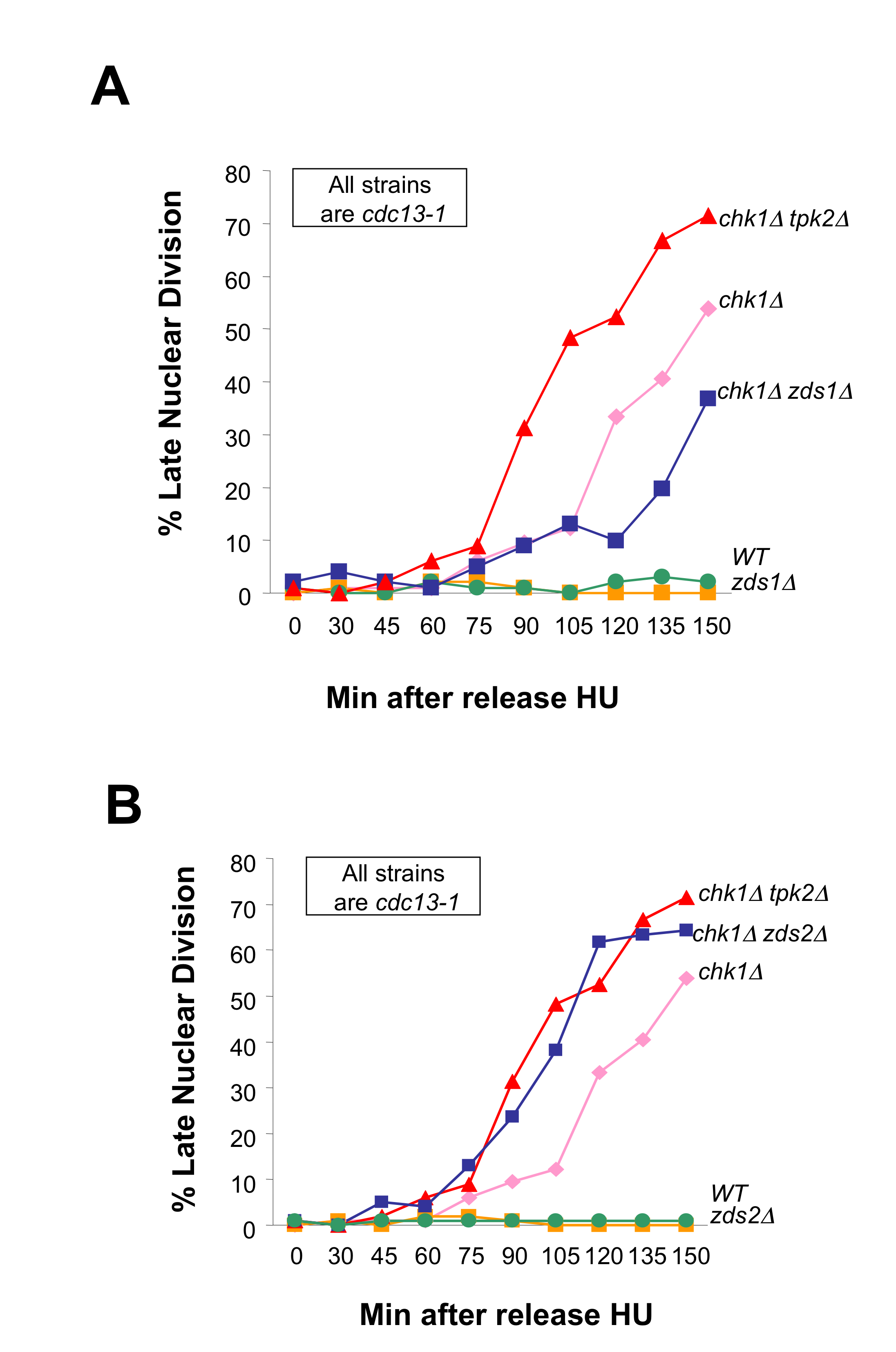

Supplement: Figure S1 — Zds2 has a role in the DNA damage checkpoint. A) cdc13-1, cdc13-1 chk1Δ, cdc13-1 zds1Δ, cdc13-1 chk1Δ zds1Δ and cdc13-1chk1Δ tpk2Δ cells were arrested in S-phase using HU and released into the cell cycle at 32°C as in Figure 2B. Aliquots from each culture were taken at indicated times following release into the cell cycle, fixed and analyzed as in Figure 2B. B) cdc13-1, cdc13-1 chk1Δ, cdc13-1 zds2Δ, cdc13-1 chk1Δ zds2Δ, and cdc13-1 chk1Δ tpk2Δ cells were arrested in S-phase using HU and released into the cell cycle at 32°C as in Figure 2B. Aliquots from each culture were taken at indicated times following release into the cell cycle, fixed and analyzed as in Figure 2B. These two graphs represent data from one experiment, therefore the controls cdc13-1 chk1Δ and cdc13-1 chk1Δ tpk2Δ are the same in both graphs. (TIF) [file pgen.1002176.s001.tif]

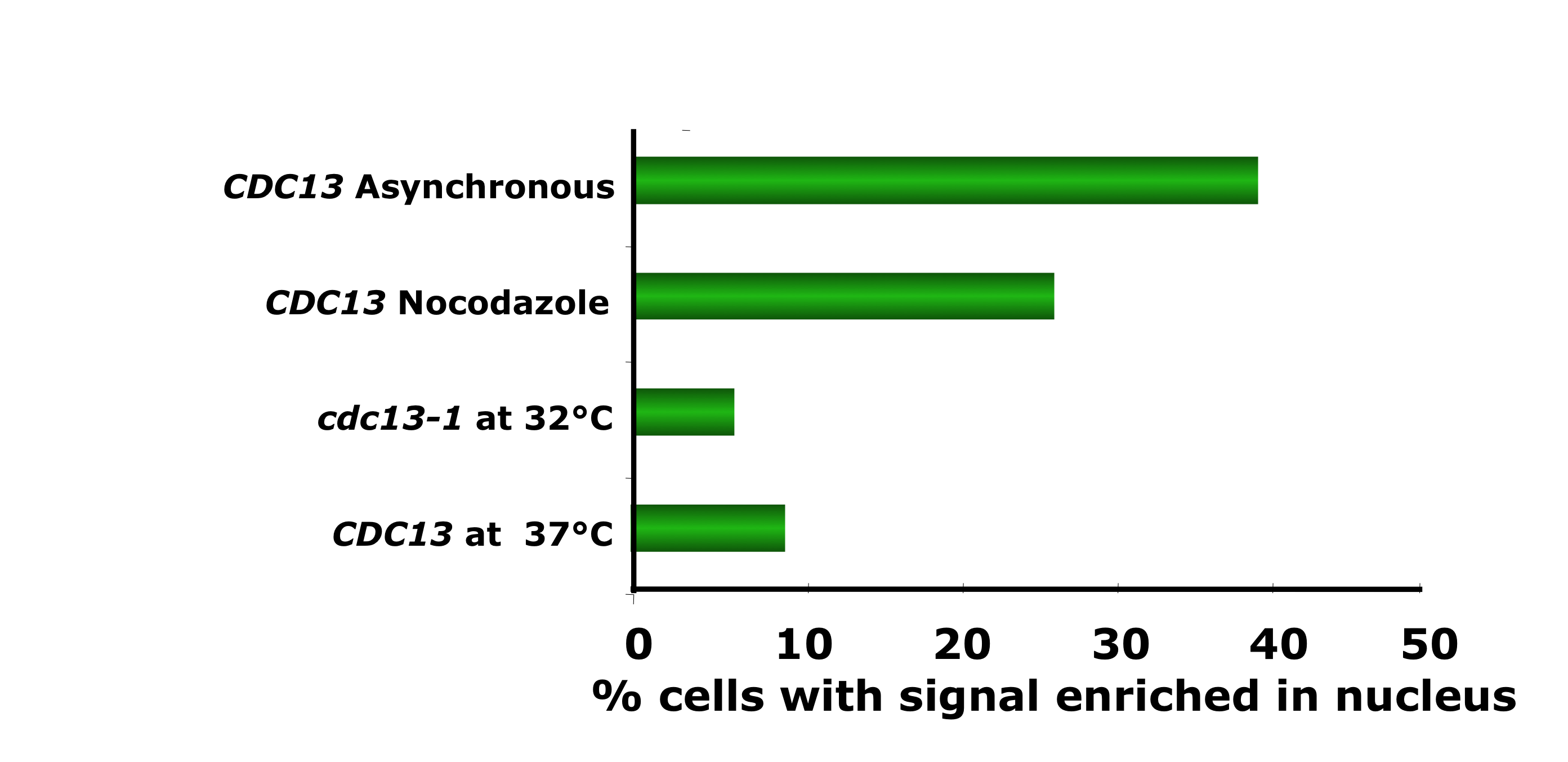

Supplement: Figure S2 — R subunit re-localization following DNA damage. WT BCY1-GFP cells and cdc13-1 BCY1-GFP cells were grown at 22°C and raised to 32°C for 2 hours. 10 µg/ml nocodazole was added to the cells as indicated for the 2 hours the cells were at 32°C. To induce heat shock, WT BCY1-GFP cells were grown at 22°C and raised to 37°C for 3 hours. To visualize GFP expression, cells were spotted on slides pre-coated with polylysine (Poly-L-lysine) and allowed to dry. GFP expression was analyzed using a Zeiss LSM 510 Meta confocal microscope. Graph represents average of two experiments. (TIF) [file pgen.1002176.s002.tif]

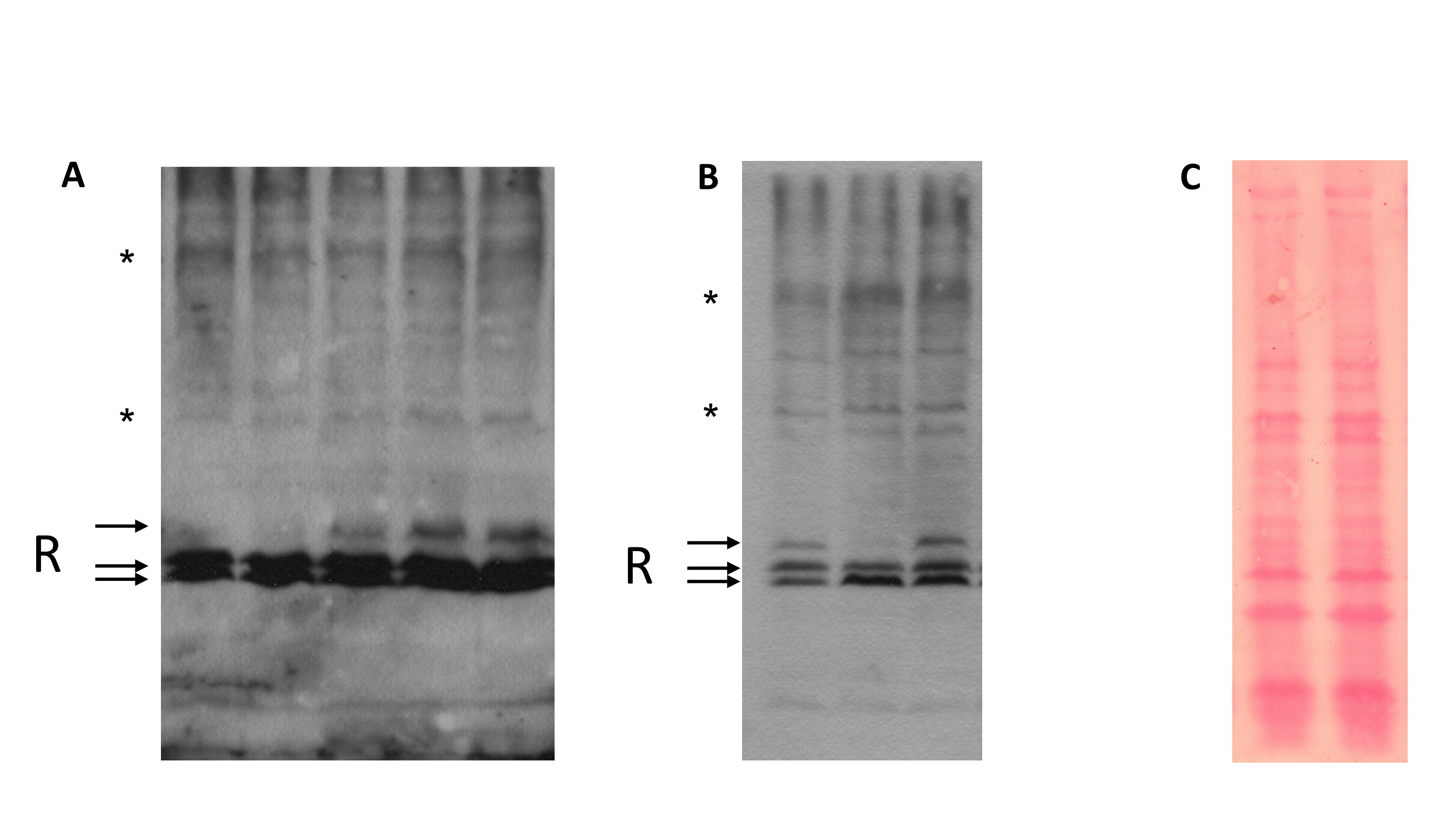

Supplement: Figure S3 — Loading controls for Figure 1. A) Entire lanes for blot shown in Figure 1A to show cross-reacting bands (*) as loading controls. B) Entire lanes for blot shown in Figure 1C to show cross-reacting bands (*) as loading controls. C) Entire lanes of Western blot of Bcy1 shown in Figure 1D stained with Ponceau S to show protein loading. (TIF) [file pgen.1002176.s003.tif]

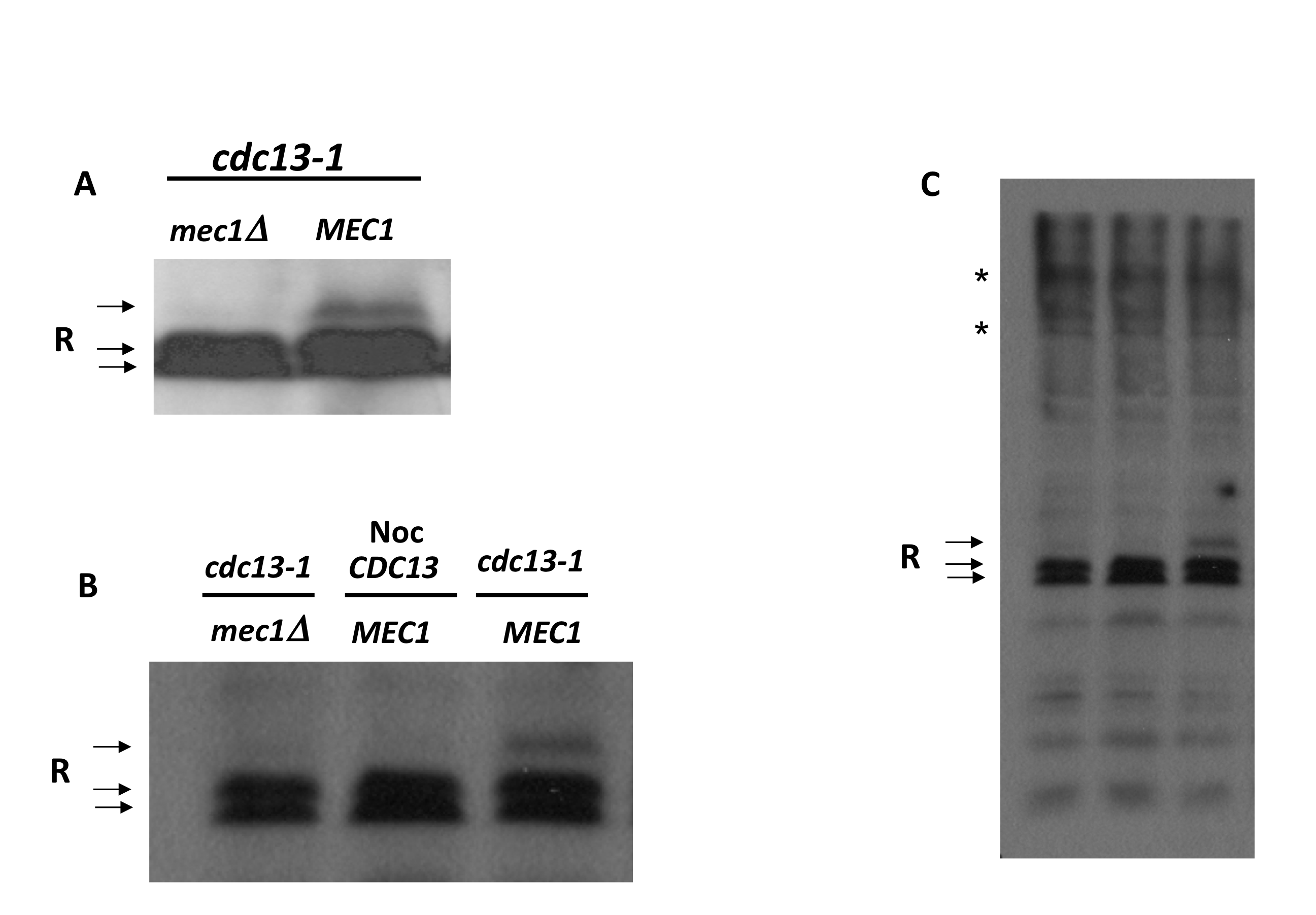

Supplement: Figure S4 — Replicate experiments showing dependence of DNA damage-induced mobility shift of Bcy1 (R subunit) on the checkpoint kinase Mec1. A) Replicate experiment to that shown in Figure 1D. cdc13-1 and cdc13-1 mec1Δ cells were grown and treated as in Figure 1B, and detection of R subunit was carried out as in Figure 1A. B) Replicate experiment to those shown in Figure 1D and (A). cdc13-1 and cdc13-1 mec1Δ cells were grown and treated as in Figure 1B, and CDC13 cells were incubated in Nocodazole as in Figure 1C. Detection of R subunit was carried out as in Figure 1A. C) Entire lanes for blot shown in (B) (here) to show cross-reacting bands (*) as loading controls. R = Bcy1. (TIF) [file pgen.1002176.s004.tif]

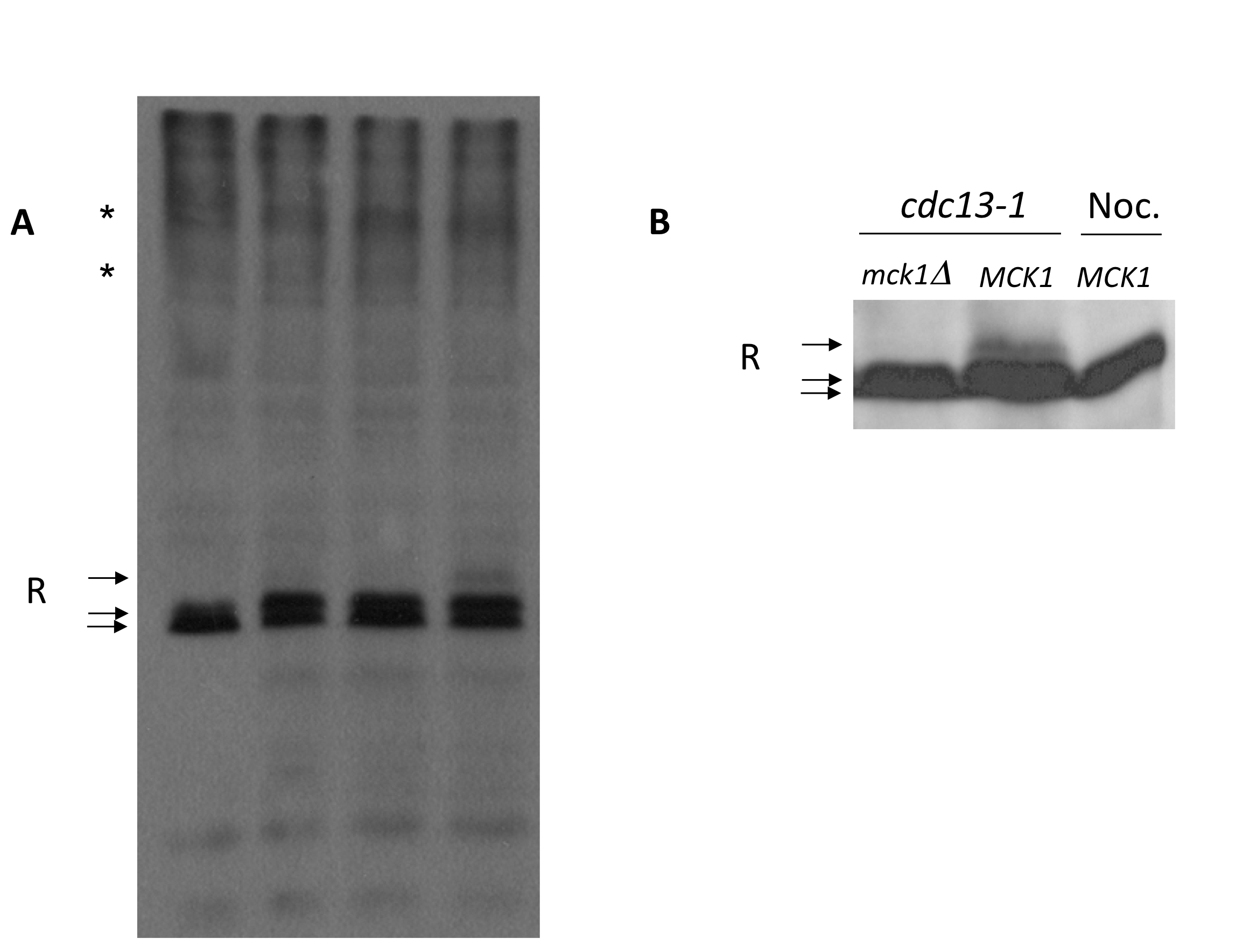

Supplement: Figure S5 — Loading control for Figure 2A and replicate experiment showing dependence of DNA damage-induced mobility shift of Bcy1 (R subunit) on the kinase Mck1. A) Entire lanes for blot shown in Figure 2A to show cross-reacting bands (*) as loading controls. B) Replicate experiment to that shown on Figure 2A. cdc13-1 MCK1 (WT), and cdc13-1 mck1Δ cells were raised to 32°C for 120 min. Nocodazole was added to MCK1 cells for 120 min. Cells were lysed, and the R subunit was detected as described in Figure 1. (TIF) [file pgen.1002176.s005.tif]

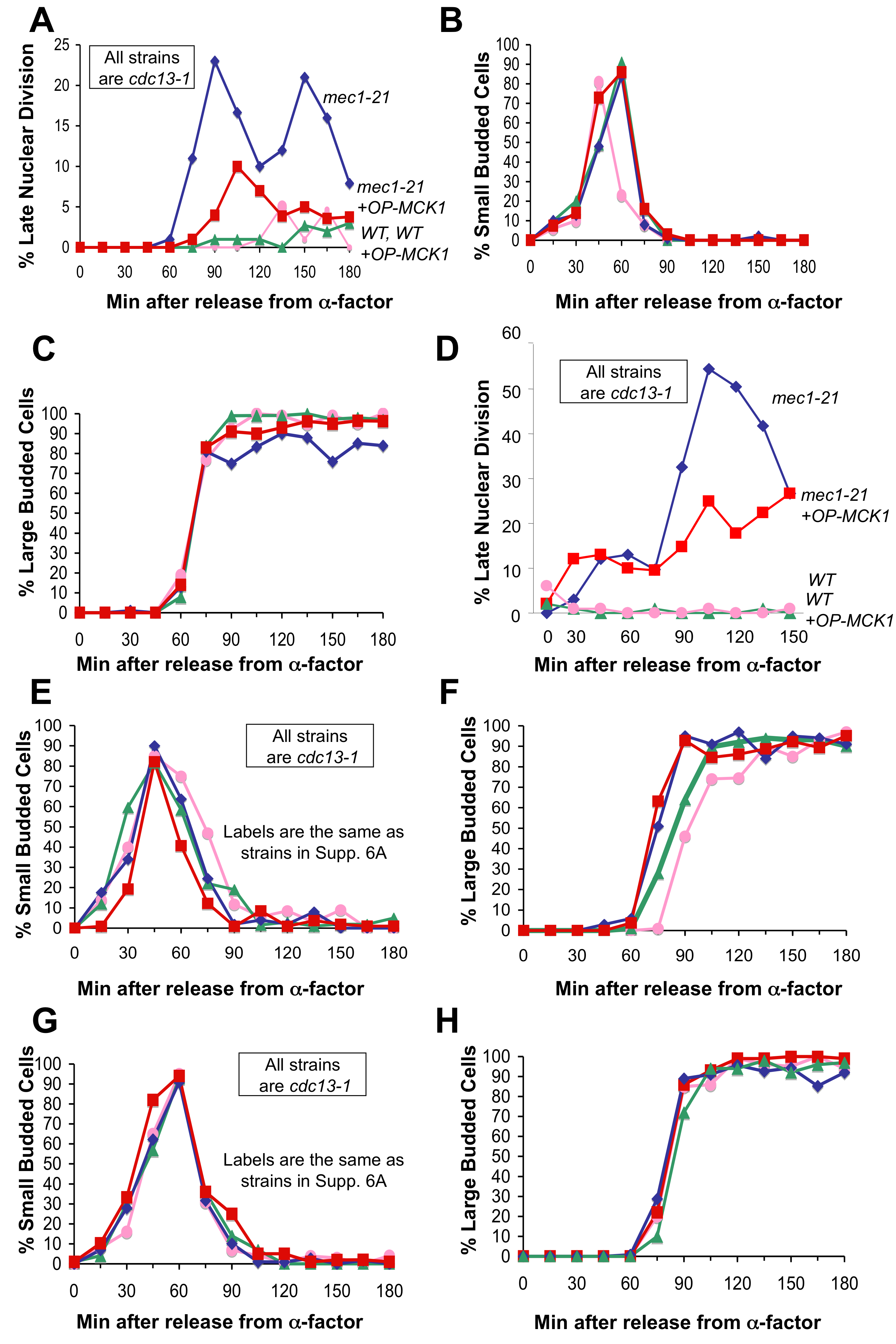

Supplement: Figure S6 — Mck1 overexpression partially alleviated the checkpoint defect of mec1-21 cells following DNA damage. A) cdc13-1 and cdc13-1 mec1-21 cells transformed with either the high-copy vector pRS425 or MCK1 in pRS425 (OP MCK1) were grown at 22°C and arrested in G1 by addition of α-factor. The temperature was raised to 32°C and the cells were released from G1 into the cell cycle at 32°C. Cells were collected at the indicated time points and analyzed as in Figure 2B. B) Cells from A were monitored for the appearance of small buds. C) Cells from A were scored for the time it took to reach large budded state with an undivided nucleus. D) cdc13-1 and cdc13-1 mec1-21 cells transformed with either the high-copy vector pRS425 or MCK1 in pRS425 (OP MCK1) were grown at 22°C and arrested in G1 by addition of α-factor. The temperature was raised to 32°C and the cells were released from G1 into the cell cycle in YPD at 32°C. Cells were collected at the indicated time points and analyzed as in Figure 2B (Replicate of Figure 2D and part A here). E) cdc13-1 and cdc13-1 mec1-21 cells transformed with either the high-copy vector pRS425 or MCK1 in pRS425 (OP MCK1) were grown at 22°C and arrested in G1 by addition of α-factor in SC -Leu. The temperature was raised to 32°C and the cells were released from G1 into the cell cycle at 32°C in SC -Leu. Cells were collected at the indicated time points and scored for the appearance of small budded cells. F) Cells from (E) were scored for the time it took to reach large budded state with an undivided nucleus. G) and H) Replicate of experiment shown in (E,F). (TIF) [file pgen.1002176.s006.tif]
